# A Comparative Study Between Ultrasound-Guided Genicular Nerve Block Combined With Interspace Between the Popliteal Artery and the Capsule of the Posterior Knee Block versus Adductor Canal Block in Total Knee Replacement

**DOI:** 10.1155/anrp/8937826

**Published:** 2025-05-22

**Authors:** Marwa M. Abouseeda, Mohamed Mohsen Rashed, Mostafa M. Hussein, Riham F. Nady, Ahmad M. Ehab

**Affiliations:** ^1^Department of Anesthesia, Intensive Care and Pain Medicine, Faculty of Medicine, Benha University, Benha, Egypt; ^2^Department of Anesthesia, Intensive Care and Pain Medicine, Faculty of Medicine, Ain Shams University, Cairo, Egypt; ^3^Department of Anesthesia, Intensive Care, and Pain Management, Faculty of Medicine, Ain Shams University, Cairo, Egypt; ^4^Department of Anesthesia, Intensive Care and Pain Medicine, Faculty of Medicine, Suez University, Suez, Egypt

**Keywords:** adductor canal block, genicular nerve block, IPACK block, knee osteoarthritis, total knee arthroplasty

## Abstract

**Background:** Total knee arthroplasty (TKA) is a surgical intervention that relieves patients experiencing severe pain and joint dysfunction.

**Objective:** The aim is to evaluate ultrasound-guided genicular nerve block (GNB) paired with infiltration between the popliteal artery and the capsule of the posterior knee IPACK block in comparison with adductor canal block (ACB) regarding the analgesia effectiveness and postoperative functional outcomes and rehabilitation parameters for TKA.

**Methods:** This randomized controlled trial enrolled 50 individuals of both genders with American Society of Anesthesiologists Class I–III, planned for TKA with spinal anesthesia. The patients were randomly allocated into two groups of 25 each. Group A underwent an ultrasound-guided GNB combined with an IPACK block, while Group B received an ACB. The amount of morphine consumed postoperatively during the initial 48 h was the main outcome. Additional outcomes encompassed postoperative knee range of motion (ROM), straight leg raising (SLR), and time up and go (TUG) test.

**Results:** Group A patients exhibited significantly lower pain perception scores at 6 and 12 h (*p* < 0.001) and lower 48 h morphine dose in comparison to Group B (*p* < 0.001). Group A had significantly better results in ROM and TUG tests on the first and second days (*p* < 0.001). No significant difference was observed in patients achieving SLR on Day 1 (*p*=0.999). Overall, Group A had a faster recovery regarding ROM and TUG and better SLR by Day 2. Group A had better satisfaction.

**Conclusions:** In TKA, combined IPACK and GNB offer superior postoperative analgesia, reduced opioid use, and improved functional outcomes compared to ACB.

**Trial Registration:** ClinicalTrials.gov identifier: NCT06423339

## 1. Introduction

Chronic knee osteoarthritis (OA), a prevalent condition in older adults, stands as a major contributor to chronic pain worldwide [1]. Total knee arthroplasty (TKA) accounts for a surgical intervention designed to alleviate patients experiencing significant discomfort and joint dysfunction due to advanced knee OA, particularly when standard nonsurgical treatments have proven ineffective [2].

Knee pain caused by OA is recognized as a separate risk factor for premature death. Consequently, minimizing postoperative pain and promoting early movement are essential strategies to reduce early mortality, prevent the development of chronic pain, and limit the reliance on opioids [3].

Enhanced understanding of knee innervation enhances postoperative TKA pain management. Aspects requiring further consideration are femoral and sciatic nerve branches that innervate the anterior and medial parts of the knee and the posterior knee joint, respectively. Historically, femoral nerve block (FNB) has remained a cornerstone of multimodal pain management for TKA patients because of its strong analgesic effect and low associated risks [4].

Nevertheless, FNB is accompanied by declined quadriceps' motor strength, discouraging physical therapy engagement, delayed mobility, and increased hospital stays for patients. In contrast, the adductor canal block (ACB) has become an excellent substitute [4].

Infiltration between the popliteal artery and capsule of the posterior knee (IPACK) block, a recent regional anesthesia method, has demonstrated a potential superior alternative for motor-sparing pain relief, offering analgesia to the knee's posterior compartment without inducing motor weakness that would impact mobility [5].

A genicular nerve block (GNB) has recently received increased interest as a novel technique for treating chronic knee pain and postoperative pain after TKA. This technique provides a satisfactory anesthetic effect for the anterior knee capsule, as well as the knee's medial and superolateral regions. Initially introduced by Choi et al., GNB, along with radiofrequency ablation of genicular nerves, was proposed as a therapeutic option for chronic knee OA. It was quickly applied to patients who undergo TKA because its efficiency in reducing pain and improving function is well established [5].

## 2. Patients and Methods

This randomized controlled trial took place over six months in the combined assembly operating theaters at Ain-Shams University Hospitals, Cairo, Egypt, between 10 May 2024 and 30 November 2024. The study included participants aged 40–70 years, classified with an American Society of Anesthesiologists (ASA) physical status of I–III, of both sexes, and planned for unilateral total knee replacement.

**Figure 1:**
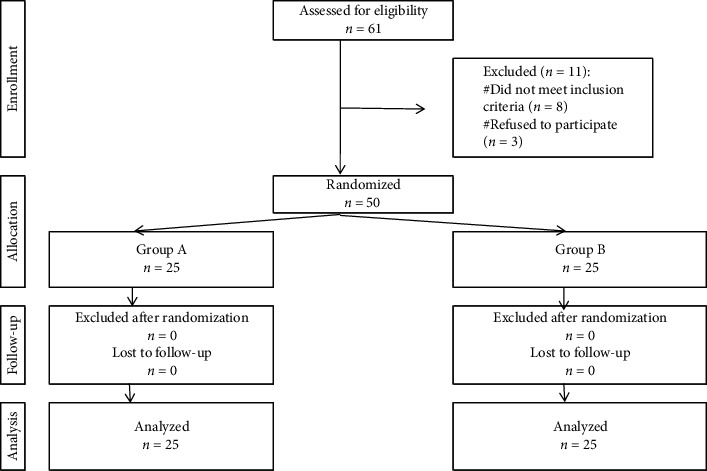
Flowchart of the cases studied.

Exclusion criteria encompassed spinal malformations, liver or renal impairment, hypersensitivity to any of the study drugs, neuromuscular or coagulopathy disorders, and patients with a history of prior knee surgery or trauma or those scheduled for revision knee arthroplasty. Fifty participants were enrolled and randomized into 2 groups, 25 each (Figure 1).

Ethical approval was obtained (approval number: FAMSU R80/2024).

Each participant provided a written informed consent form after being thoroughly informed about the study's objectives, potential risks, and advantages prior to enrollment in the study.

Personal information was handled confidentially, and participation was completely optional, allowing individuals the freedom to withdraw at any time. Confidentiality of all participant information was assured.

Through computer-generated randomization, participants were allocated to one of two groups. An independent data coordinator managed the allocation process using sealed, sequentially numbered opaque envelopes accessible only to the anesthesiologist performing the blocks.

Group A underwent an ultrasound-guided GNB combined with an IPACK block, while Group B (control) received an ACB.

The study design compared two active interventions (IPACK + GNB vs. ACB) rather than including a “no block” group. This decision was likely made because both techniques are used in clinical practice, and it would be unethical to deny regional anesthesia to patients undergoing painful surgery such as TKA.

Before surgery, all participants completed a detailed history and clinical evaluation, accompanied by routine laboratory investigations. They were instructed to fast for 8 h from solid foods and 2 h from clear liquids. Upon entering the operating room, intravenous access was obtained, and Ringer's acetate was administered at 10 mL/kg. Baseline monitoring involved noninvasive blood pressure (NIBP), electrocardiography (ECG), and arterial oxygen saturation (SpO_2_). Pain intensity was appraised using the numerical rating scale (NRS), and preoperative training was provided for all participants. Spinal anesthesia was performed using a 25G or 27G needle while the patient was seated, targeting the intervertebral space between L3 and L4 or L4 and L5, delivering 2.5–3 mL of 0.5% hyperbaric bupivacaine. The sensory block aimed to achieve a level at or above the 10th thoracic dermatome. Hypotension, characterized by a ≥ 20% reduction from baseline blood pressure, was treated with intravenous ephedrine (6 mg boluses) [6].

Nerve blocks were administered by a single, highly experienced regional anesthesiologist not affiliated with the study, ensuring consistency and eliminating potential performance bias.

**Figure 2:**
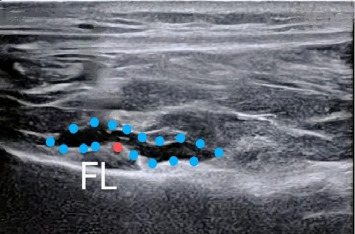
Ultrasound-guided superomedial genicular nerve block. Red circle: genicular artery, blue dotted line: spread of local anesthetic, and FL: femoral line.

In Group A, following skin closure, 15 mL of 0.25% bupivacaine with adrenaline (1:400,000) was administered. The GNB was performed using an ultrasound probe with a high frequency (10–15 MHz) to identify the genicular nerves, inferior medial, superior lateral, and superior medial, near their respective arteries (Figure 2). After confirmation of the genicular arteries via Color Doppler, a 5 mL volume of anesthetic was administered at each target site [6]. The IPACK block was then performed by visualizing the popliteal artery and the distal femur posterior aspect. A 20G 80 mm needle was placed in a medial-to-lateral direction, aligned with the femur, and 20 mL of anesthetic was administered incrementally [7].

**Figure 3:**
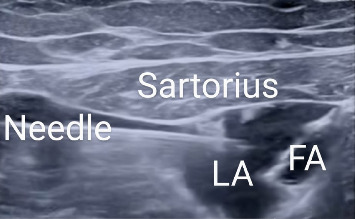
Ultrasound-guided adductor canal block. FA: femoral artery and LA: local anesthetic.

In Group B, the adductor canal was located utilizing a linear ultrasound probe (6–13 MHz) by visualizing the superficial femoral artery in a cross-sectional plane. The ACB was performed at the mid-thigh level under ultrasound guidance, where the femoral artery lies beneath the sartorius muscle, and the saphenous nerve is typically visualized adjacent to the artery. Local anesthetic was deposited adjacent to the femoral artery, deep to the sartorius muscle. The nerve to the vastus medialis and the medial femoral cutaneous nerve were not specifically targeted. A 22G 80 mm needle was guided in-plane, moving from the lateral to the medial direction, and 20 mL of 0.5% bupivacaine was administered under sterile conditions, ensuring periarterial spread (Figure 3). TKA was then performed by skilled surgeons using a minimally invasive mini-midvastus technique along with manual cement mixing methods [8]. A tourniquet is applied to the thigh and inflated throughout the surgery to minimize blood loss and improve the surgical field visibility. The choice of prosthesis (whether ligament cutting or ligament preservation) was based on patient anatomy, ligament integrity, and surgeon preference.

Pain intensity was evaluated using NRS at 0, 6, 12, 24, and 48 h following the operation. The time to the initial demand for rescue analgesia (intravenous morphine 3 mg), defined as NRS > 3 [8], was also recorded Functional outcomes were evaluated through straight leg raising (SLR), scored on a scale from 0 (*absence of muscle contraction*) to 5 (*normal strength when resisting force*). The timed up and go (TUG) assay, which assessed functional mobility by timing how quickly patients could stand from a seated position, cover a 3-m distance, turn around, return, and sit back down. Patients were encouraged to mobilize within 24 h postoperatively when motor strength reached a score of 2 [8]. Quadriceps motor strength was assessed via SLR [6]. A self-reported scale (satisfied, somewhat satisfied, somewhat dissatisfied, and dissatisfied) was employed for patient satisfaction assessment; we used a simple satisfaction scale for ease of interpretation, and adverse effects were documented [8].

Postoperative analgesia consisted of intravenous paracetamol (1 g per 6 h) and intravenous ketorolac (30 mg per 12 h). Pain at rest was monitored using the NRS (scored 0–10, with mild pain rated 1–3, moderate pain 4–7, and severe pain at 8 or above), along with the need for rescue analgesia. The study's endpoint included the evaluation of outcomes up to 48 h postoperatively.

The main outcome was the overall morphine amount used in the initial 48 h following surgery. Additional outcomes comprised postoperative functional recovery and rehabilitation metrics, such as quadriceps' motor strength assessed via SLR, knee range of motion (ROM), and TUG test performance.

The first 48 h are critical in postoperative pain management, as they influence opioid use, early mobilization, and functional recovery.

The sample size calculation relies upon the findings of Et et al., 2021 [8], which reported a mean of total morphine consumption in the intervention group as 197 ± 61.8, while in the control group as 380 ± 83, with 5% alpha error and an 80% study power; the necessary sample size is 50 participants, divided into 25 per group. The sample size calculation was accomplished with STATA Version 10. The sample size was calculated based on morphine consumption, which was the primary outcome. While this provides statistical power for assessing opioid use, it does not ensure adequate power for secondary outcomes such as ROM, TUG, or pain scores. To enhance generalizability, future studies should use larger sample sizes and power calculations based on multiple outcome measures. However, given resource constraints, the current study still provides valuable preliminary data that can guide larger trials.

The gathered data were coded, tabulated, and analyzed using IBM SPSS statistics software Version 28.0, IBM Corp., Chicago, United States of America, 2021. For quantitative data, the mean ± standard deviation (SD) was used, and comparisons were made utilizing the independent *t*-test. Qualitative data were expressed as counts and percentages, with comparisons accomplished utilizing the chi-square test and Fisher's exact test. The log-rank test was applied to evaluate the rate of first morphine dose request. Statistical significance was set at a *p* value of ≤ 0.050; *p* values exceeding this level were considered insignificant.

## 3. Results

Table 1 compares the baseline attributes of the studied groups (A and B), showing no significant differences in age, BMI, sex distribution, ASA classification, or operation duration (all *p* > 0.05). This indicates that both groups are similar.

Table 2 compares the pain perception and morphine analgesia requirements between Group A and Group B. Pain perception scores at Hours 6 and 12 were significantly declined in Group A than in Group B (*p* < 0.001), with a mean difference of −3.8 ± 0.3 and −3.7 ± 0.2, respectively. However, no significant differences were observed at Hours 0, 24, and 48 (*p* > 0.05). Regarding morphine requirements, Group A had a significantly longer time to the first dose (15.6 ± 2.8 h vs. 7.5 ± 2.4 h, *p* < 0.001) and a substantially lower total 48-h dose (6.1 ± 2.4 mg vs. 31.3 ± 6.7 mg, *p* < 0.001). These findings suggest that Group A experienced better pain control and required less morphine compared to Group B.

Table 3 shows that Group A had significantly better results in ROM and TUG tests on the first and second days (*p* < 0.001), indicating faster mobility and joint function recovery. Nevertheless, no significant difference was noticed in the proportion of participants achieving SLR on Day 1 (*p*=0.999). On Day 2, Group A had a higher proportion of successful SLRs than Group B (*p*=0.024). Overall, Group A had a faster recovery regarding ROM and TUG on Day 1 and Day 2 and better SLR by Day 2.

Table 4 reveals no significant difference in side effects between Groups A and B, with similar rates of hypotension and no cases of bradycardia or anaphylaxis. However, Group A had significantly higher satisfaction, with 76.0% of participants satisfied compared to 40.0% in Group B (*p*=0.001). The relative risk for satisfaction in Group A was 1.90, indicating a higher likelihood of satisfaction in Group A. Overall, while side effects were comparable, Group A had better satisfaction.

## 4. Discussion

Knee pain associated with OA is a separate risk factor for early mortality, emphasizing the importance of reducing postoperative pain and promoting early mobilization. Regional nerve blocks are critical for mitigating early mortality, alleviating long-term pain, and minimizing the reliance on opioids [3].

This study is dedicated to comparing the efficiency of various nerve blocks in postoperative pain relief, functional outcomes, and knee rehabilitation following TKA. Our findings showed that Group A (IPACK + GNB) experienced significantly lower pain perception than Group B (ACB alone) at 6 and 12 h postoperatively. In addition, Group A had a longer time to the first morphine dose and a reduced total 48-h morphine consumption.

Functional outcomes, including ROM, TUG test, and the SLR test, were significantly better in Group A during the first two postoperative days. Notably, no significant differences in side effects that include hypotension, bradycardia, or anaphylaxis were found between groups. Group A had significantly increased patient satisfaction.

The ACB primarily supplies analgesia to the anterior capsule of the knee but does not adequately address posterior knee pain. To overcome this limitation, the IPACK block has become a promising regional anesthesia approach. This motor-sparing approach affords good analgesia of the knee's posterior quarter without compromising walking ability. Likewise, the GNB has been described as a new technique for treating chronic and postoperative knee pain through targeting the anterior capsule and medial and superolateral aspects. The motor-sparing characteristics of GNB enable early mobilization, enhanced physiotherapy, and earlier discharge [5].

Complications such as hematoma, nerve injury, or local anesthetic toxicity are rare, requiring larger studies to assess incidence accurately. No serious complications were observed, but due to the small sample size, the study is underpowered to detect rare events.

We agree that the addition of an IPACK block to an ACB has been shown to enhance posterior knee analgesia, and we recognize the growing consensus around this combination in clinical practice. However, the primary objective of our study was to evaluate the analgesic efficacy of GNB as an alternative strategy to improve postoperative pain control in TKA, particularly in terms of posterior knee pain, which is not well-covered by ACB alone.

Our rationale for including the IPACK block only in the GNB group assumed that both GNB and IPACK would work synergistically to address the anterior and posterior components of knee pain. This combined approach was intended to serve as an alternative to traditional ACB–based strategies, rather than to isolate the effect of GNB alone.

Et et al. [8] mentioned that using an IPACK block with the ACB enhances postoperative pain control, reduces opioid consumption, improves functional outcomes, and lowers hospital stay. Similarly, Eid et al. demonstrated that GNB is a reliable and efficient substitute to periarticular injection for managing postoperative pain in TKA. Although periarticular injection required less morphine in the first two postoperative days, its analgesic effect diminished after 18 h, necessitating rescue analgesia during rehabilitation. In contrast, GNB's pain scores began to increase between 10 and 12 h postoperatively, requiring rescue analgesia at this time.

In a systematic review and meta-analysis, Guo et al. [9] documented that incorporating an IPACK block reduced postoperative visual analog scale (VAS) scores at rest and during activity. They also noted that the postoperative morphine requirement was lesser, and the activities of daily living were performed better, the total distance walked was also more, and the length of stay was shorter. Notably, patients receiving the combined IPACK + ACB exhibited superior TUG test performance and postoperative ROM than those receiving ACB alone, with no significant differences in quadriceps' muscle strength.

Kampitak et al. [10], in a randomized clinical trial, compared the pain-relieving impacts of IPACK, GNB, and the combination of IPACK + GNB, all used alongside continuous ACB in TKA patients. Their findings specified that the combination of IPACK + GNB offered notably better pain relief during movement at 4 and 8 h after surgery compared to each block administered separately. This combination also improved pain control during the initial 24 h, likely due to its multimodal approach. The analgesic effects of IPACK and GNB were most pronounced within the first 12 h postoperatively, consistent with our findings.

Conversely, in their prospective randomized trial, Abdullah et al. [11] evaluated the effects of the addition of an IPACK block to ACB versus ACB solely. They reported comparable TUG test and SLR outcomes at 12 and 24 h postoperatively. These findings differ from our results because our study included GNB, which provided superior pain control and motor function, leading to more effective physiotherapy, shorter hospital stays, and higher patient satisfaction; also, possible reasons may include different anesthetic concentrations/doses, variability in patient demographics or baseline function, and different physiotherapy protocols.

## 5. Conclusion

In total knee replacement surgery, combined IPACK and GNB offer superior postoperative analgesia, reduced opioid use, and improved functional outcomes compared to ACB. Benefits include better knee ROM and TUG test performance at 24 and 48 h postoperatively, better quadriceps strength (SLR) at 48 h postoperatively, along with greater pain relief and patient satisfaction.

### 5.1. Limitations

This study has several limitations. Reliance on ultrasound-guided techniques requires experienced operators. The small sample size (50 patients) may limit the generalizability of the findings, and the absence of a nonintervention control group prevents assessing the absolute efficacy of the regional blocks. The short follow-up period (48 h) does not capture long-term pain control, opioid use, or functional recovery. A longer follow-up period (e.g., 1 week, 1 month, and 3 months) would help evaluate chronic pain, long-term functional outcomes, and complications.

Although no major complications were observed, the small sample size may not detect rare adverse events. A larger multicenter study is recommended to improve safety assessment.

Patient satisfaction was assessed using a simple scale rather than a validated tool (e.g., PROMIS Pain Interference Score, VAS for satisfaction), introducing potential bias. Future research should address these limitations by increasing sample size, extending follow-up, including a standard-care control group, and using validated assessment tools to improve reliability and clinical relevance.

## Figures and Tables

**Table 1:** Comparison according to demographic characteristics.

Variables		Group A (total = 25)	Group B (total = 25)	*p* value
Age (years)		60.8 ± 7.4	59.8 ± 7.9	0.621 ^

Sex (*n*, %)	Male	14 (56.0%)	15 (60.0%)	0.774^#^
	Female	11 (44.0%)	10 (40.0%)	

BMI (kg/m^2^)		28.7 ± 2.7	29.3 ± 3.3	0.461 ^

ASA (*n*, %)	I	8 (32.0%)	6 (24.0%)	0.812^#^
	II	12 (48.0%)	13 (52.0%)	
	III	5 (20.0%)	6 (24.0%)	

Operation duration (minutes)		95.2 ± 13.6	96.0 ± 10.8	0.802 ^

*Note:* Data presented as mean ± SD or number (%).

Abbreviations: ASA =  American Society of Anesthesiologists; BMI = body mass index.

^ Independent *t*-test.

^#^Chi-square test.

**Table 2:** Comparison regarding pain perception and the need for morphine analgesia.

Variables	Group A (total = 25)	Group B (total = 25)	*p* value	Relative effect	
				Mean ± SE	95% CI
Pain perception (NRS-10)					
Hour 0	0.3 ± 0.5	0.5 ± 0.5	0.257 ^	−0.2 ± 0.1	−0.4–0.1
Hour 6	0.6 ± 0.8	4.3 ± 1.5	**< 0.001** ^**∗**^ ^	−3.8 ± 0.3	−4.4–−3.1
Hour 12	0.6 ± 0.7	4.2 ± 0.9	**< 0.001** ^**∗**^ ^	−3.7 ± 0.2	−4.1–−3.2
Hour 24	5.9 ± 1.1	6.4 ± 1.5	0.205 ^	−0.5 ± 0.4	−1.2–0.3
Hour 48	3.7 ± 1.0	4.0 ± 1.2	0.324 ^	−0.3 ± 0.3	−1.0–0.3
Need for morphine analgesia					
Time to first dose (hour)	15.6 ± 2.8	7.5 ± 2.4	**< 0.001** ^**∗**^ ^	8.1 ± 0.7	6.6–9.6
Total 48 h dose (mg)	6.1 ± 2.4	31.3 ± 6.7	**< 0.001** ^**∗**^ ^	−25.2 ± 1.4	−28.1–−22.4

*Note:* Data are presented as mean ± SD unless mentioned otherwise. Relative effect: effect in Group A is relative to that in Group B. The bold values represent statistically significant differences between Group A and Group B.

Abbreviations: CI = confidence interval; SE = standard error.

^ Independent *t*-test.

^∗^Significant.

**Table 3:** Comparison regarding knee's functional outcomes.

Variables	Group A (total = 25)	Group B (total = 25)	*p* value	Relative effect	
				Mean ± SE/relative risk	95% CI
ROM (degrees)					
Day 1	92.9 ± 6.2	86.1 ± 4.2	**< 0.001** ^**∗**^ ** ^**	6.8 ± 1.5	3.8–9.8
Day 2	100.6 ± 2.5	90.3 ± 3.5	**< 0.001** ^**∗**^ ** ^**	10.3 ± 0.9	8.6–12.0
TUG (seconds)					
Day 1	40.0 ± 2.4	46.3 ± 2.3	**< 0.001** ^**∗**^ ** ^**	−6.3 ± 0.7	−7.6–−5.0
Day 2	33.6 ± 2.4	40.6 ± 2.6	**< 0.001** ^**∗**^ ** ^**	−6.9 ± 0.7	−8.3–−5.5
SLR					
Day 1					
IV	23 (92.0%)	24 (96.0%)	0.999^§^	0.96	0.83–1.10
V	2 (8.0%)	1 (4.0%)		Reference	Reference
Day 2					
IV	15 (60.0%)	22 (88.0%)	**0.024** ^**∗#**^	0.68	0.48–0.97
V	10 (40.0%)	3 (12.0%)		Reference	Reference

*Note:* Data are presented as mean ± SD or number (%) unless mentioned otherwise. Relative effect: effect in Group A is relative to that in Group B. The bold values represent statistically significant differences between Group A and Group B.

Abbreviations: CI = confidence interval; SE = standard error.

^Independent *t*-test.

^#^Chi-square test.

^§^Fisher's exact test.

^∗^Significant.

**Table 4:** Comparison regarding side effects and patients' satisfaction.

Variables	Group A (total = 25)	Group B (total = 25)	*p* value	Relative effect	
				Relative risk	95% CI
Side effects					
Hypotension	4 (16.0%)	5 (20.0%)	0.999^§^	0.80	0.24–2.64
Bradycardia	0 (0.0%)	0 (0.0%)	NA	NA	NA
Anaphylaxis	0 (0.0%)	0 (0.0%)	NA	NA	NA
Satisfaction					
Very satisfied	19 (76.0%)	10 (40.0%)	**0.001** ^**∗§**^	1.90	1.12–3.22
Fair (somewhat satisfied or somewhat dissatisfied)	5 (20.0%)	3 (12.0%)		Reference	
Very unsatisfied	1 (4.0%)	12 (48.0%)			

*Note:* Data are presented as number (%) unless mentioned otherwise. Relative effect: effect in Group A is relative to that in Group B. The bold value represents statistically significant differences between Group A and Group B.

Abbreviations: CI = confidence interval; NA = not applicable; SE = standard error.

^§^Fisher's exact test.

## Data Availability

The data that support the findings of this study are available from the corresponding author upon reasonable request.
